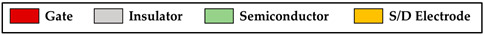# Split-Gate: Harnessing Gate Modulation Power in Thin-Film Electronics

**DOI:** 10.3390/mi15010164

**Published:** 2024-01-22

**Authors:** Subin Lee, Yeong Jae Kim, Hocheon Yoo

**Affiliations:** 1 Department of Electronic Engineering, Gachon University, Seongnam 13120, Republic of Korea; 2 Korea Institute of Ceramic Engineering and Technology, Ceramic Total Solution Center, Icheon 17303, Republic of Korea

**Keywords:** split-gate, thin-film transistor, neuromorphic device, logic circuit, light-emitting device, photodetector, high-gain amplifying device

## Abstract

With the increase in electronic devices across various applications, there is rising demand for selective carrier control. The split-gate consists of a gate electrode divided into multiple parts, allowing for the independent biasing of electric fields within the device. This configuration enables the potential formation of both p- and n-channels by injecting holes and electrons owing to the presence of the two gate electrodes. Applying voltage to the split-gate allows for the control of the Fermi level and, consequently, the barrier height in the device. This facilitates band bending in unipolar transistors and allows ambipolar transistors to operate as if unipolar. Moreover, the split-gate serves as a revolutionary tool to modulate the contact resistance by controlling the barrier height. This approach enables the precise control of the device by biasing the partial electric field without limitations on materials, making it adaptable for various applications, as reported in various types of research. However, the gap length between gates can affect the injection of the electric field for the precise control of carriers. Hence, the design of the gap length is a critical element for the split-gate structure. The primary investigation in this review is the introduction of split-gate technology applied in various applications by using diverse materials, the methods for forming the split-gate in each device, and the operational mechanisms under applied voltage conditions.

## 1. Introduction

Due to the distinct attributes inherent in diverse materials, electronic devices have been intricately tailored to specific device types that align with the unique properties of these materials. Considerable research and development efforts have been invested in the exploration of thin-film transistors (TFTs), utilizing a diverse array of thin-film materials. Various materials, including metal oxides characterized by low power consumption and minimal leakage [1,2,3,4], transition metals dichalcogenides (TMDs) exhibiting high stability and tunable bandgap [5,6,7], carbon nanotubes (CNTs) with notable conductivity and diverse structures [8,9], and organic semiconductors characterized by biocompatibility and scalability to various device types [10,11,12,13], have been employed in the construction of electronic devices. These TFTs offer remarkable versatility in terms of substrate compatibility, with applications extending to transparent glass [14,15], banknotes [16,17], paper [18,19], skin [20,21], and flexible plastics [22,23,24]. Their utility extends to a wide spectrum of domains, encompassing light-emitting devices [25,26] neuromorphic computing [27,28,29], optoelectronic devices [30,31,32], gas sensors [33,34], biosensors [35,36], and security [37,38].

Typically, TFTs function with a solitary gate that spans the entire channel region, extending from the source to the drain [39,40,41,42,43]. The gate electric field serves as the key determinant of channel conductivity. The application of a negative or positive voltage bias to the gate regulates the injection of holes and electrons, facilitating both *p*-channel and *n*-channel field-effect transistor (FET) operations [44,45,46]. Unlike typical TFTs that operate with a single gate, devices that use multi-gates to implement various operations are emerging. As a type of device that operates as a multi-gate, split-gate devices have been reported. In contrast to the conventional TFT structure, a novel avenue emerges by subdividing the gate into two or more smaller gates, commonly referred to as the “split-gate” configuration [47,48,49,50,51,52]. This innovative approach permits the selective application of voltages to the split-gates, enabling the localized introduction of electric fields within the channel. This, in turn, empowers more precise control over charge carriers, enabling the selective manipulation of holes and electrons [53,54,55,56], as well as the induction of hole–electron recombination [57,58], thereby offering a spectrum of additional functionalities.

Early split-gate research focused on the structural characteristics of split-gates and mainly explored their electrical properties, but the scope of application gradually expanded. Since the inception of the concept [47], diverse applications have emerged, as illustrated in Table 1 [59,60,61,62,63]. In alignment with this perspective, we present an extensive examination of the split-gate structure and its diverse applications in TFTs. Firstly, we delve into the operational principles and their significant contributions to TFTs. Subsequently, we scrutinize the realm of split-gate-based logic operations, with particular emphasis on reconfigurable logic. Thirdly, we explore the deployment of split-gate structures in neuromorphic applications. Moving on to the fourth aspect, we reevaluate the role of split-gate structures in light-emitting transistors, showcasing their capability in the precise control of charge carriers, both holes and electrons. In the fifth section, we delve into the use of split-gates in phototransistors, highlighting the advancements in photo-sensitivity. Sixth, we focus on the versatility of split-gate operations in multi-modal applications and amplifiers. Lastly, we offer our insights and outlook regarding the split-gate technology and its potential future applications.

## 2. Discussion

### 2.1. Operation Principle

In conventional TFTs, the channel is formed when a voltage is applied to the gate, and the operation is based on the carrier transports between the source and drain. In contrast, the split-gate structure, which features multiple gates instead of the conventional single gate, enables the precise control of the device by applying either identical or distinct voltages to the gates. This adjustment allows for the modulation of channel conductivity in response to the applied voltage. The split-gate device exhibits the capability to selectively enhance or impede carrier transport through the partial electric field, dependent on the polarity of the voltage bias. In particular, the electric field distribution is significantly influenced by the gate gap length. Therefore, this section includes an illustrative example depicting variations in channel conductivity depending on the gate gap lengths.

In this section, we will introduce diverse applications of split-gate technology across various materials. These applications include the adjustment of the Fermi level, the control of carrier injection through a split-gate structure, and the utilization of split-gates to mitigate contact resistance. Notably, this split-gate technology can be applied to any material without limitations. Consequently, we aim to elaborate on the formation of the gate gap based on the material and discuss the operation at the fabricated device.

**Figure 1:**
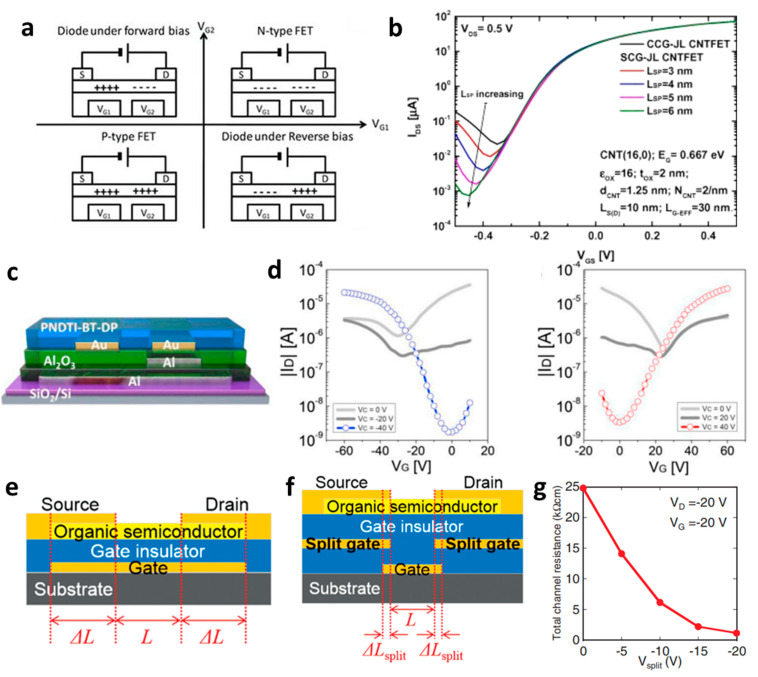
(**a**) Variation in electric field inside the channel according to biasing two gate voltages [64] (adapted from [64] with permission from WILEY−VCH Verlag GmbH & Co. KGaA, Weinheim, Germany). (**b**) Decreased off−current trend according to gap length adjustment [65] (adapted from [65] with permission from Elsevier B.V.). (**c**) Schematic diagram of a device with split-gate inserted vertically, and the transfer curve of it which (**d**) counter−carrier is prevented by split-gate unipolar operation [66] (adapted from [66] with permission from Elsevier B.V.). (**e**) Transistor of conventional structure, (**f**) reduction of overlap distance between gate and s/d by inserting split-gate, and (**g**) total channel resistance by voltage applied to split-gate [68] (adapted from [68] with permission from WILEY−VCH Verlag GmbH & Co. KGaA, Weinheim, Germany).

The split-gate structure facilitates the independent control of carrier injection in ambipolar semiconductor devices. Hsu et al. introduced a device incorporating a blend of poly[N-9′-hepta-decanyl-2,7-carbazole-alt-5,5-(4′,7′-di-2-thienyl-2′,1′,3′-benzothiadiazole)] (PCDTBT) and [6,6]-phenyl C_70_-butyric acid methyl ester (PC_70_BM), which are commonly employed ambipolar materials in bulk heterojunction composites [64]. In this bulk ambipolar system, PCDTBT and PC_70_BM serve as a hole- and electron-transporting polymer, respectively. Upon their combination in bulk, it was observed that they functioned as a *p*-channel FET with a hole-dominated conduction channel. To achieve reconfigurable ambipolar operation, equal polar voltages are applied to G1 and G2, and the diode operates when opposite polar voltages are applied. As depicted in Figure 1a, *V*_G2_ was swept from −100 V to 100 V, and voltage conditions of −100, −50, −25, 0, and 25 V were applied to *V*_G1_. Consequently, it was verified that the device operates as a unipolar *n*-channel FET when a positive voltage larger than zero is applied, facilitating the transport of electrons through the gates and the sweeping gate. With the application of higher voltages, carrier accumulation takes place at the semiconductor/dielectric interface, enabling operation in the n-channel mode and resulting in an increased drain current (*I*_D_).

In addition to bulk materials, our investigation extended to research where split-gate was employed with CNTs and polymers that exhibit ambipolar properties as single materials. Naturally, CNTs with zig-zag structures inherently possess ambipolar characteristics.

Tamersit et al. constructed a transistor employing CNTs and observed a noteworthy enhancement of the ambipolar behavior attributed to the band-to-band tunneling (BTBT) mechanism [65]. The electrical characteristics of the device utilizing the split-gate were investigated by altering the gate gap length, as illustrated in Figure 1b. The device exhibited that with the increase in the gate gap length to 6 nm, BTBT decreased, gate leakage current decreased, the off current reduced, approaching unipolar operation, and the on/off ratio demonstrated an increasing trend.

In another research endeavor, an ambipolar polymer was employed. While several reports exist on ambipolar organic devices functioning under high vacuum or N_2_ conditions, as exposure to ambient air can degrade electron transport in ambipolar organic materials. However, Yoo et al. successfully fabricated a stable device with a bottom gate/bottom contact structure even in ambient air. The achievement was realized through the utilization of the poly{[N,N′-bis(3-decylpentadecyl)-naphtho[2,3-b:6,7-b′]dithiophene-4,5,9,10-tetracarboxidiimide-2,7-diyl]-alt-5,5′-(2,2′-bithiophene)} (PNDTI-BT-DP) as the semiconductor [66]. Additionally, they proposed the integration of a dielectric layer to achieve a targeted gap size between the main gate and the secondary gate in the split-gate structure. This approach was found to be more effective in controlling the gap size compared to the lateral structured split-gate. To attain gate logic operation using a single ambipolar material, a balanced *p*- and *n*-channel FET operation is imperative. In this paper, the device exhibited the effect of screening the electric field at the main-gate electrode by inserting the control gate between the main-gate and drain electrode, as depicted in Figure 1c. This strategy aimed to achieve a well-balanced carrier operation. Hence, the ambipolar semiconductor could function as a unipolar transistor, as demonstrated in Figure 1d. The counter carrier was effectively suppressed, blocking the electric field between the control gate and the main gate, leading to the drain electrode when an identical polar voltage was applied. This resulted in the realization of well-balanced reconfigurable ambipolar characteristics, with parameters such as *μ*_h_ = 8.3 × 10^−3^ cm^2^ ∙V^−1^∙s^−1^, *μ*_e_ = 8.0 × 10^−3^ cm^2^ ∙V^−1^∙s^−1^, *V*_to,h_ = *V*_to,e_ = 0 V, *V*_th,h_ = −17.7 V, and *V*_th,e_ = 22.2 V, due to the additional gate bias at the control gate.

In addition to TFT, the split-gate configuration has also found application in gate-tunable diodes. Hughes et al. utilized the split-gate structure and asymmetric contact electrodes to manipulate the characteristics of a single-walled carbon nanotube (SWCNT)-based diode [67]. In particular, when asymmetric electrodes were employed, the device demonstrated pronounced rectifying characteristics with low leakage current in the absence of any gate biasing. Nevertheless, the introduction of a gate into this device led to a reversal of CNT diode operation, resulting in variable leakage current based on the gate bias. With a positive drain bias and negative gate bias applied to the device, the energy barrier increased, obstructing electron injection from the electrode while permitting the possibility of hole injection, the minority carrier, at the Pd contact. Even with a negative gate bias, when a positive bias was applied, the energy barrier for hole injection was higher at the Ti contact. Consequently, the device could operate as a unipolar transistor with an enhanced on/off ratio, achieved by reducing counter carrier transport, especially for holes, due to the higher energy barrier at the Ti contact.

Lots of organic transistors are pursued to fabricate the short channel device for high performance. However, efforts to reduce the contact resistance are essential, as the contact resistance often surpasses the channel resistance. Uemura et al. successfully decreased the contact resistance by minimizing parasitic capacitance through the adoption of the split-gate [68]. In general, reducing the length of overlap between the gate and source/drain (s/d) electrode is beneficial for high-speed operation. However, a challenge arises as the contact resistance increases with the reduced overlapped length, constrained by the limitation of the charge injection in conventional planar transistors (Figure 1e). The adoption of the split-gate mitigates this issue, as the overlapping length between the split-gate and s/d electrode does not contribute to the input capacitance. The use of the split-gate electrode enables the attainment of a larger carrier injection area, with the overlapping length between the main gate electrode and the split-gate electrode solely contributing to the parasitic capacitance, as shown in Figure 1f. Hence, a device incorporating the split-gates, where the electrode is positioned below the s/d electrode, can facilitate the high-speed operation by achieving a high cut-off frequency with low contact resistance due to the minimization of the overlapped length (Figure 1g). Thus, the split-gate configuration can be integrated into both planar and vertical structures, with ongoing efforts focused on reducing the gap size through the structure modifications. Importantly, the split-gate technology is versatile and can be applied to various materials and structures, showcasing its potential to fabricate high-performance electronic devices through selective carrier injection and a reduction in contact resistance.

### 2.2. Logic Operation

The split-gate allows for the selective carrier. In this section, the operation of the gate will be introduced by independently controlling several gates independently for the logic operations.

**Figure 2:**
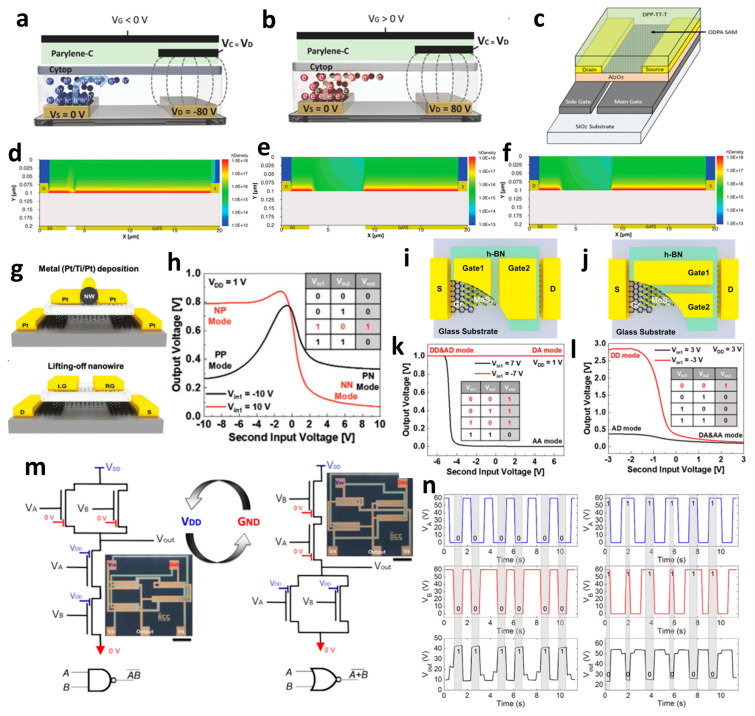
(**a**) *p*−channel, (**b**) *n*−channel unipolar FET characteristics that operate according to the electric field conditions applied by introducing a split-gate in a bipolar operating channel [69] (adapted from [69] with permission from WILEY−VCH Verlag GmbH & Co. KGaA, Weinheim). (**c**) Asymmetric gate electrode size for well−balanced operation of the carrier. Gaps with lengths of (**d**) 1 μm, (**e**) 2 μm, and (**f**) 6 μm exist to verify the carrier operation behavior by gap size in the split-gate [70] (adapted from [70] with permission from WILEY−VCH Verlag GmbH & Co. KGaA, Weinheim). (**g**) Gap produced by nanowire lift−off to implement a sub−μm length gap, (**h**) inverter operation implemented according to voltage conditions in the device [71] (adapted from [71] with permission from Wiley−VCH GmbH). Device schematic diagram according to the arrangement direction of split-gate located (**i**) horizontally to s/d and (**j**) vertically to s/d. And then the inverter operation result: split-gate located (**k**) horizontally to s/d and (**l**) vertically to s/d [72] (adapted from [72] with permission from Springer Nature). (**m**) Schematic diagram of *V*_DD_ and GND connections for making reconfigurable circuits and (**n**) their transient measurement at NAND and NOR circuits [73] (adapted from [73] with permission from Springer Nature.

The conventional OTFT split-gate exhibits significant hysteresis attributed to the shallow traps from the hydroxyl group in the gate insulator such as SiO_2_ and Al_2_O_3_, commonly used for high-performance devices. However, Yoo et al. developed a device suitable for logic operations with minimal hysteresis and reduced gate bias stress. They achieved this by employing the CYTOP and Parylene-C dielectric layer [69]. In general, both holes and electrons can operate in ambipolar devices. However, the type of transport carrier can be determined by the bias condition of the control gate in the split-gate transistor, as shown in Figure 2a,b. When the size of the control gate is designed to be larger than the drain electrode, the counter carrier of the drain electrode can be suppressed completely. Both hole and electron operation can be achieved by increasing the off current through the injection of the counter carrier from the *I*_D_ at the voltage of the control gate (*V*_C_) = 0 V. However, when *V*_C_ is applied to be 80 V (−80 V) to enable unipolar transistor operation by preventing the counter carrier, the unipolar *n*-channel (*p*-channel) FET operation becomes available, because the gate electrode can suppress the transportation of the counter carrier. This device can operate as a configurable unipolar transistor, and it can evolve into a complementary inverter due to the minimal hysteresis. Unlike the conventional inverter, which exhibits the Z-shaped voltage transfer characteristics (VTCs) due to the leakage current, the split-gate transistor utilized in the inverter shows a full swing due to the lower leakage current. Additionally, the DC gain and output swing are larger than those of the conventional inverter.

Also, to be fabricated into the complementary system, Yoo et al. constructed the split-gate inverter by designing the different dimensions for the split-gate, as shown in Figure 2c [70]. The side gate was designed to be shorter than the length of the main gate, as its primary function is to block the injection of the counter-carrier from the drain. When the device operates as an *n*-channel (*p*-channel) TFT, a positive (negative) bias equal to or greater than the drain voltage must be applied to the side gate to prevent an unwanted counter carrier or hole (electron) injection at the drain electrode. In this paper, carrier injection by gap size was observed, as shown in Figure 2d–f. If the length exceeds 2 μm, the carrier cannot transport the channel, so the inverter was fabricated with a gap length of 2 μm. When a drain voltage (*V*_DD_) = 0 V was applied to the conventional inverter, the bottom (top) device did not completely turn off, due to the hole (electron) counter-carrier. In contrast, when *V*_DD_ = 0 V was applied to the split-gate adopted inverter, it exhibited a complete turn-off operation when the side gate was set to the 0 V, blocking the hole in the *n*-channel FET operation, and blocking the electron in the *p*-channel FET operation.

Likewise, a short-length gap is required, because the carrier cannot transport if the gap size exceeds a certain limit. Various studies have aimed to shorten the gap size, and Lee et al. fabricated a laterally structured gate-tunable PN diode [71]. To create a gate-tunable diode, electrodes are deposited. Before gate deposition, a nanowire is positioned in the center of the targeted location, as shown in Figure 2g. The 200 nm gap length, which is the length of the nanowire, can be achieved by lifting off the nanowire after evaporating the gate electrode. The variation of the inner junction was observed by the applied voltage at two gates, showcasing the controllable operation of the gate. PdSe_2_ TMD was used in this device. This semiconductor implements different junctions (PP, PN, NP, and NN) by biasing the voltage differently. The device was biased with a voltage of −6 V (6 V) on the gate targeting *p*-channel (*n*-channel) operation. The split-gate transistor can generate the wanted junction, which can bend the band independently, by modulating the Fermi level to control the wanted counter carrier. To operate the inverter, the *V*_DD_ was fixed to 1 V, and the gate voltage was biased at two split-gates. A total of −10 V was applied for the input ‘0’, and 10 V was applied for the input ‘1’. Therefore, this device can operate the SAND gate, because the state ‘1’ output occurs only in the NP type, which operates the $A\bar{B}$, as shown in Figure 2h.

The TMD-based logic gate paper was reported by Lee et al., demonstrating the NAND and NOR gates based on the arranged direction of the split-gate by using the MoS_2_ [72]. There are two structures: one where two split-gates are in series, similar to two homogeneous transistors in the same direction as the s/d arrangement, as shown in Figure 2i, and the other structure is located perpendicular to the s/d electrode arrangement, as shown in Figure 2j. In the structure where two homogeneous transistors are connected in parallel, applying a negative bias to either of the two gates prevents electrons’ accumulation and operation. However, when both positive biases are applied, a channel is formed, operating in depletion mode, and turning on. In a structure as in Figure 2j, when a positive bias is applied to one of the two gates, the channel can be partially turned on, forming only a small portion of the channel. However, if a negative bias is applied to the two gates, the device does not operate, because the MoS_2_ cannot form the channel. Hence, carrier concentration can be independently controlled by biasing the voltage at each split-gate. The inverter was operated using the split-gate for the drive TFT and the resistor for the pull-up operation. The device was measured by biasing the voltage at two gates. The fixed voltage was applied at *V*_1_, and the voltage range was swept at *V*_2_. When negative bias (A mode) was applied, it was set to ‘0’, and when positive bias (D mode) was applied, it was set to ‘1’. As a result, when two gates exist, similar to series-connected TFTs, they do not operate except under the condition that positive bias is applied to both devices. Therefore, the resistor is completely pulled up, and a value of ‘1’ outputs. When a positive bias is applied to all gates, the drive TFT operates linearly, resulting in a pull-down after 0 V. Consequently, the output is ‘0’, and NAND operates as shown in Figure 2k. On the other hand, when two gates exist in parallel, a negative bias is applied to the two gates to turn them off, so ‘1’ outputs because this is the point where the resistance is pulled up. In the transistor operation under AD conditions, it is turned on but has a very low value; so, the resistance is not completely pulled up, and the value converges to ‘0’. In the case of DA and AA modes, the transistor operates, so a pull-down occurs after 0 V, and the state is ‘0’, so the NOR gate could operate.

Another example involves the utilization of organic materials to implement NOR and NAND gates. Yoo et al. developed a vertically structured split-gate for this purpose [73]. Instead of fabricating separate circuits for implementing NAND and NOR functions, they achieved a complementary logic in a reconfigurable method by simply altering the connections of *V*_DD_ and GND. In this study, they utilized the poly[{2,5-bis(2-hexyldecyl)-2,3,5,6-tetrahydro-3,6-dioxopyrrolo[3,4-c]pyrrole-1,4-diyl}- alt -{[2,2′:5′,2′′-terthiophene]-5,5′′-diyl} (PDPP3T) ambipolar material, known for its excellent hole-transporting properties, rather than electron transport. The introduction of the split-gate structure resulted in the formation of an electric field, extending even to the edge of the gate, facilitating carrier accumulation. In the case of *n*-channel FET operation, it was observed that electrons were effectively trapped and completely accumulated within the gap area, whereas, in the case of *p*-channel FET operation, holes were weakly accumulated and were not confined solely to the central area of the gap. In this paper, the authors conducted a study to compare circuit operations between conventional circuits, co-planar structures, and non-planar structures based on the structure of the device. As a result, the non-planar structure allowed the channel to form continuously, owing to the sub-*μ*m thin gap which corresponds to the thickness of the dielectric. Consequently, thanks to its structural advantages, it achieved the maximum hole and electron *I*_D_, resulting in higher output swing, noise margin, and gain, when compared to other structures. If the split-gate is adopted in an ambipolar semiconductor device, unwanted charge injection can be prevented when the drain voltage is applied, allowing the NAND circuit to be electrically reconfigured into a NOR circuit and vice versa. This reconfiguration depends on the voltage applied to the side gate and can be achieved simply by altering the connections of GND and *V*_DD,_ as shown in Figure 2m. The transient measurement at NAND and NOR circuits is depicted in Figure 2n. This study represents the first instance of complementary logic circuits fabricated using ambipolar organic transistors, achieved by effectively suppressing the counter carrier through the use of ambipolar materials.

A study has been conducted to implement seven logic operations by dividing the gate operation range with TMD-based materials. Ying et al. utilized tellurium nanoribbons for this purpose [74]. The device structure featured tellurium (Te) as the semiconductor, an insulating layer of hexagonal boron nitride (h-BN) on top, and a split-gate with a 440 nm gap created through non-invasive scanning probe lithography. By introducing a split-gate to the ambipolar material, the internal semiconductor can be configured as an NP or PN junction, depending on the voltage applied to each gate. When a positive voltage is applied to the gate, it results in *n*-doping, while a negative voltage applied to the gate led to *p*-doping. A voltage range of −11 V to 4 V was applied to the back gate, while a fixed voltage was applied to the two split-gates. Specifically, +6 V represented a logical ‘1’, and −6 V served as ‘0’ input. A high-level output was defined as exceeding 10^−10^ A, while a low-level output was set as less than 10^−13^ A. The role of the gate operation varied depending on whether the transistor functioned as a pull-up or pull-down device. When a resistor was utilized as the pull-up element, the circuit functioned as the OR, XOR, and NAND gates based on the voltage range. Conversely, when the resistor was employed as the pull-down element, the circuit operated as the AND, NOR, and XNOR gates. Additionally, when identical inputs were applied to both gates and the resistor was used for the pull-up element, the circuit functioned as the NOT gate. Overall, the Te split-gate FET was demonstrated to operate all seven different logic gates.

In the work by Bestelink et al., a structure similar to a split-gate, referred to as a multimodal transistor (MMT), was utilized. The MMT featured an additional gate on the source side, known as the source gate, in addition to the primary gate [75]. The source gate was divided into two parts and positioned perpendicular to the s/d. The gap between the source gates and control gate was 1 μm. In this configuration, the source gate, especially the part overlapping with the source electrode, modulated charge injection. The accumulation of charge in the channel resulted in a pinch-off at the source side, leading to saturation at low drain voltage and flat output characteristics. Based on this principle, limitations arise in the transfer characteristics controlled by the control gate when the channel resistance is lower than the source injection area. In this paper, we utilized three MMTs and a load resistance to implement the XNOR logic gate. The circuit configuration involved two transistors and a pull-up resistor. M1, a three-gate device with a control gate, included source gates divided into two parts oriented perpendicular to the s/d electrodes. The OR operation was performed by applying inputs A and B to the two parallel source gates in the respective device. On the other hand, M2, a transistor with a control gate and a single source gate, not split source gates, executed the NAND operation between inputs A and B, utilizing the signal generated in M1 as the gate. Finally, by connecting M1 to the load resistance *R*_L_ and inverting the output through an inverter operation, we achieve the XNOR operation, which can operate to $\left(A\cdot B\right)+\left(\bar{A}\cdot \bar{B}\right)\equiv \left(A\cdot B\right)+\bar{A+B}\equiv \bar{(A+B)\cdot \bar{A\cdot B}}$. Consequently, the XNOR operation is performed, and the XOR function is also easily implemented by negating the drain voltage. In this way, there is also the possibility of implementing a low-voltage-based XNOR using a MMT as a unipolar device.

### 2.3. Neuromorphic Device

Conventional neuromorphic devices typically exhibit large dimensions and high-power consumption, necessitated by the extensive interconnections between devices required to emulate the complexity of the human brain. To address these challenges, the split-gate configuration has been employed to enable the selective operation of the neuromorphic devices through independent gate modulation.

**Figure 3:**
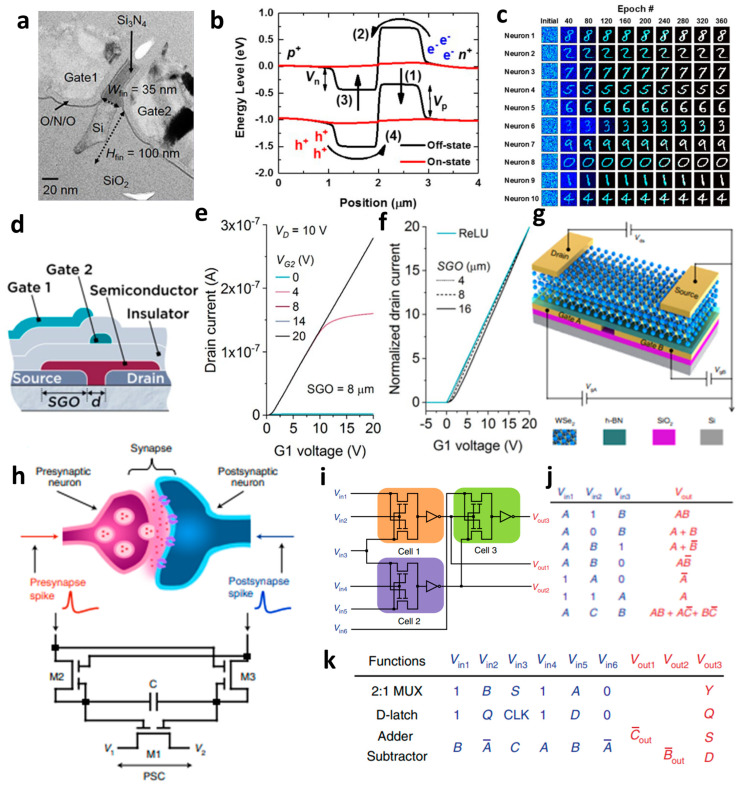
(**a**) Split-gate FET in FinFET structure with a 35 nm length split-gate gap, (**b**) schematic diagram of carrier injection barrier control according to voltage biasing at the split-gate, (**c**) MNIST test results on a neuromorphic device that operates by inducing charge trapping in the feedback system generated by split-gate device operation according to the number (#) of epochs [63] (adapted from [63] with permission from Frontiers Media S.A.). (**d**) Schematic diagram of a multi-modal transistor that operates similarly to a split-gate, (**e**) transfer curve at each gate voltage application condition, (**f**) neuromorphic operation of ReLU function implementation according to gap size adjustment [78] (adapted from [78] with permission from Springer Nature). (**g**) Schematic diagram of a split-gate device using ambipolar TMD WSe_2_, (**h**) schematic diagram of neuromorphic operation implemented in a circuit of three transistors using split-gate, (**i**) gate operation in an extended circuit. (**j**) Gate operation in one cell, and (**k**) gate operation implemented in the extended cell [79] (adapted from [79] with permission from Springer Nature).

To implement the sub-*μ*m length gap, Choi et al. utilized the Si_3_N_4_ as the spacer, in the structure of SiO_2_/Si_3_N_4_/blocking SiO_2_ [63]. They fabricated a FinFET-type split-gate by utilizing the n^+^ poly-Si as the gate with the fin body length of 35 nm (Figure 3a). The energy barrier undergoes variations due to rapid increases in the current induced by the positive feedback (PF), triggered by *V*_G1_ (gate 1). With an increase in *V*_G1_, the electron injection barrier decreases, allowing electrons to be injected into the hole injection barrier, thereby reducing the hole injection barrier. Consequently, by injecting the holes at the electron injection barrier, the electron injection barrier is further diminished, and the PF loop continues through successive injections of electrons at the hole injection barrier, as shown in Figure 3b. With the repetition of this sequence, the device can swiftly transition from the turn-off to turn-on state. As a signal is repeatedly transmitted from the synapse of the PF device to G1, it accumulates in the charge trap layer of the PF device, leading to a gradual decrease in the *V*_on_ of the PF. Consequently, the PF device is designed as a neuromorphic device which rapidly switches from the turn-off to the turn-on state when the *V*_on_ falls below the *V*_read_. During the pattern recognition simulation utilizing the synapse array and PF neurons, the weights of 784 (28 × 28) presynaptic neurons are updated with the input signals corresponding to 10 Modified National Institute of Standards and Technology database (MNIST) numbers. The learning rule is applied, training the presynaptic neuron as shown in Figure 3c. Hence, they propose a neuromorphic device based on charge trapping, achieving a low-voltage operation of approximately ~0.25 pJ/spike through the super-steep subthreshold swing (S.S.) spike by reducing the S.S. using the method. Unlike conventional neuromorphic devices that utilize capacitance for storing carriers, this approach, detailed in this paper, enables charge accumulation without a capacitance layer by utilizing the charge trap layer.

Plasticity is essential for the adaptive structural and functional changes in the brain. Kamal et al. accomplished short-term plasticity (STP) and long-term plasticity (LTP) by fabricating the tunable-split-gate synaptic transistor (T-SGST) [76]. Split into two gates, gate 1 (G1) has only oxide under the gate, while the oxide/nitride/oxide layer is stacked under gate 2 (G2). G2 is responsible for controlling the trapping and de-trapping of holes, and G1 is utilized for switching the pulse from STP to LTP, modulating the number of repetitive input pulses. STP occurs when a hole is trapped and de-trapped in the SiGe storage node. As input pulses are applied, synaptic learning progresses as holes become trapped and de-trapped in the nitride layer. The transition from STP to LTP is accomplished through multiple input pulses. Each gate performs a specific operation and function in the T-SGST device, with each gate facilitating neuromorphic operation.

Additionally, to imitate neuron behavior, the operation of excitatory and inhibitory connections is required. Lee et al. induced excitatory and inhibitory operation by alternating the polarity of the ferroelectric layer according to the signal applied to the split-gate [77]. When a positive bias was applied to the gate, the polar direction of the ferroelectric layer was oriented in a specific direction, reducing the threshold voltage, *V*_th_, and the neuron transitioned to the excitatory state. In contrast, when a negative bias was applied to the gate, which induces inhibition, the ferroelectric layer did not change polarity or can suppress excitatory operation by altering the polarity of the ferroelectric layer under the gate. Unlike the conventional neuromorphic device of FeFET type, which is ineffective at energy consumption due to a residual current caused by the residual polarization, this approach offers improved energy efficiency. However, when an *n*-channel FET is connected to the SG FeFET, the residual current of the SG FeFET is reduced when there is no signal to the excitatory gate. Therefore, they proposed a low-power operation neuromorphic device by implementing a method where the device consumes power only when the signals are applied into the neuron, effectively eliminating the residual current.

Also, there is a report of a TFT using the two separated gates. Although Pesch et al. did not explicitly use the word ‘split-gate’, they introduced the multimodal transistor (MMT), recognized for its energy efficiency and high performance in analog and other mixed-signal applications [78]. As shown in Figure 3d, the MMT was controlled by applying gate voltage biasing, as shown in Figure 3e. Carrier injection and current switching can be independently controlled by two gates. Specifically, gate 1 (G1) modulated the current value, while gate 2 (G2) controlled channel switching after the channel had completely accumulated. This device can perform a similar operation to the rectified linear unit (ReLU) function, as shown in Figure 3f, by modulating the electrical characteristics through input voltage bias at G1 and controlling the channel switching at G2. The MMT is capable of executing neuromorphic operations related to the ReLU activation function. The ReLU function outputs ‘I2’ if the input ‘I1’ is smaller than the ‘I2’, and outputs ‘I1’ if ‘I1’ is the same or larger than ‘I2’. In neural networks, the ReLU function is employed to block a signal if the input is smaller than a certain value, and to transmit a value if the input is greater than or equal to a certain value. Therefore, the MMT device can modulate the output to a linear or exponential function based on the device structure and voltage biasing.

Pan et al. conducted the research by implementing a device capable of both the neuromorphic and logic gate operations, utilizing the split-gate transistor [79]. They introduced the electrically tunable homojunction (ETH) by adopting the tungsten diselenide (WSe_2_) as the channel material. As shown in Figure 3g, WSe_2_ and hexagonal boron nitride (h-BN) were exfoliated, and the two gate electrodes were created with the 500 nm gap length. The WSe_2_ semiconductor operates based on the polarity of the drain voltage, leveraging its ambipolar characteristic. The device applied a different polarity of the drain voltage to control the power of synapse emulation using the capacitor. The transistor with three split-gates was indicated in Figure 3h to facilitate the neuromorphic operations. M1 serves as a core computing device that processes spike signals transmitted from M2 and M3, utilizing a capacitor. The capacitor is employed to store charge and adjust the potential difference between the two split-gates of the M1 device. Upon the application of an input spike signal to M2 and M3, the capacitor undergoes electrical charging. Subsequently, when the spike signal is eliminated, the high resistance of M2 and M3 impedes the capacitor discharge, thereby maintaining a constant potential between the two gate electrodes of the M1 device. Channel doping in M1 is achieved by inducing a change in resistance. The synapse behavior is emulated through channel doping. Upon charging the capacitor, the potential difference at the capacitor varies, causing the electrical conductivity of the M1 device to increase or decrease based on the potential difference. This, in turn, results in synapse excitation and inhibition. The device operates neuromorphically, mimicking the Hebbian and anti-Hebbian learning rules by controlling the *V*_1_ and *V*_2_. This circuit enables neuromorphic behavior by emulating the continuous strengthening and weakening of synaptic connections. In this paper, the authors not only implemented operations but also incorporated logic functions, not only neuromorphic operation. The gate operation is contingent on the drain voltage, as well as the polarity and biased voltage of two gate electrodes. If the circuit is fabricated as shown in Figure 3i, the various logic functions can be implemented by different combinations of the voltage biasing. When one cell is operated as shown in Figure 3i, a total of seven combinations can be operated as shown in Figure 3j. Additionally, if the circuit is expanded to include three cells, an additional four logic gates can be implemented, as shown in Figure 3k. This paper introduced the research that operates the neuromorphic computing and reconfigurable multifunctional logic simultaneously, utilizing the TMD material.

### 2.4. Light-Emitting Device

Split-gate technology is applicable not only in computing but also in optical devices. In particular, the ability to control both electron and hole carriers becomes crucial when implementing a split-gate in a light-emitting transistor (LET).The LET can effectively regulate the current flow through the gate, enabling precise control over the location of light emission based on the quantity of electrons injected at the source.

**Figure 4:**
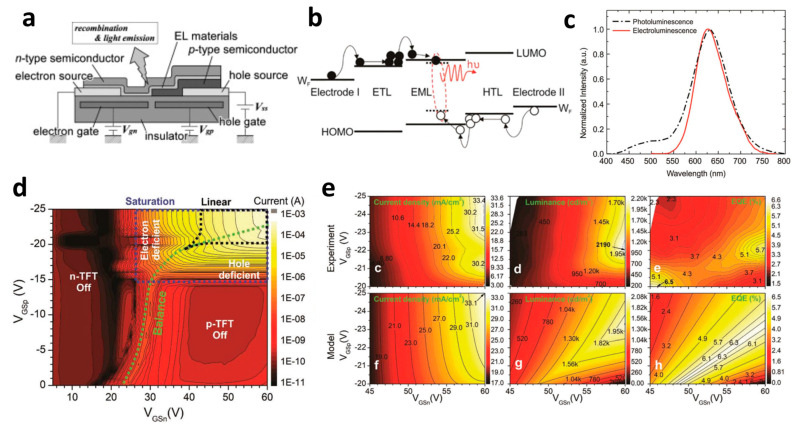
(**a**) Cross−sectional schematic of LET with split-gate electrode [61] (adapted from [61] with permission from Elsevier B.V.), (**b**) the flat band diagram where both holes (transporting at HOMO level) and electrons (transporting at LUMO level) are transported according to the application of a split-gate voltage across the layer of the structure, (**c**) PL and EL data to verify light emission wavelength. (**d**) Split-gate voltage biasing condition mapping data where the two carriers are balanced and so are their current values. (**e**) Mapping data of experimental and modeling values that evaluate current density, luminance, and EQE values, in addition to current [80] (adapted from [80] with permission from WILEY−VCH Verlag GmbH & Co. KGaA, Weinheim).

Suganuma et al. developed the organic light emitting transistor (OLET), utilizing ambipolar materials [61]. Conventional ambipolar OLET often suffers from a low on/off ratio, substantial leakage, and capacitance issues. However, the off current of this device has been significantly reduced, leading to a higher on/off ratio compared to conventional OLETs. This improvement is attributed to the independent control of electrons and holes, possibly achieved through the introduction of the split-gate OLET. As demonstrated in Figure 4a, the off current was regulated by maintaining a positive potential at the hole-gate voltage. This device effectively suppresses the counter carrier in the off state while enhancing the current, emission, and on/off ratio by activating the carrier in the on state. The introduction of the split-gate enables one to achieve an appropriate length between the electroluminescence (EL) region and the injection of electrons and holes. Parasitic resistance is induced at the carrier recombination position in OLET. As previously discussed, the split-gate can decrease parasitic resistance by ensuring an adequate length at the recombination point for electrons and holes. Consequently, the emission efficiency of OLET can be increased, leading to improved EL efficiency and reduced parasitic resistance.

Lee et al. coined the device the ‘overlapping-gates OLET’ (OG-OLET), incorporating the split-gate structure with the dielectric positioned between two gates [80]. In this configuration, Gate I functioned as the main gate, while Gate II served as the side gate, partially overlapping in the perpendicular direction. The light-emitting layer was constructed using 4-(dicyanomethylene)-2-t-butyl-6-(1,1,7,7-tetramethyljulolidyl-9-enyl)-4H-pyran (DCJTB)-doped Tris-(8-hydroxyquinoline)aluminum (Alq_3_). Electrons and holes were injected from Gate I and Gate II, as depicted in Figure 4b, and transported to the channel through their respective transport layers. Hence, the position and intensity of light emission can be modulated by controlling the injection and transportation of electrons and holes through voltage biasing at each gate. Electrons and holes transport to the center of the device, which is the light emitting area. The light is emitted in this region through the recombination of electrons and holes. When using DCJTB for the fluorescent layer, it was noted that the peak wavelength at the electroluminescence (EL) spectrum was 628 nm (Figure 4c), with a high photoluminescence (PL) quantum yield of 73 ± 2%. In contrast, a higher PL quantum yield of 86 ± 2% was observed when Alq_3_ was doped with DCJTB, attributed to the presence of a horizontal emitting dipole. The device exhibited high external quantum efficiency (EQE) at elevated brightness levels, with both brightness and EQE remaining high. The paper reported an EQE of 5.7% and a brightness of 2190 cd m^−2^. Additionally, the current value depends on the distribution and concentration of carriers influenced by the gate bias, as illustrated in Figure 4d. The experimental and simulated values of photonic characteristics in OG-OLET are depicted in Figure 4e, corresponding to the gate biasing.

Besides its application in transistor types, split-gate technology has also been employed in TMD-based light-emitting diodes. Bie et al. presented research which featured integrated circuits capable of simultaneously implementing LEDs and photodetectors [81]. In this study, an ambipolar TMD, MoTe_2_, was utilized as the active layer. They utilized split top gates with a 400 nm length gap. A MoTe_2_ transistor was employed at the split-gate to control and form the PN junction. The split-gate can control the concentration of either holes or electrons, enabling each carrier to play a dominant role in a specific region. Additionally, the doping level can be selectively adjusted by independently modulating the voltage. When the PN junction is formed by adjusting the concentration of each carrier, it leads to a distinct rectification effect within the unipolar semiconductor, resulting in the emission of light in the near-infrared (NIR) region, where the PL of MoTe_2_ occurs. The photodetector can operate when the np junction is formed, and the photocurrent can be produced when the laser wavelength of 1160 nm, which is the light absorption region, irradiates the device. The photocurrent exhibited a linear increase when the light intensity was linearly applied to the device, resulting in an EQE of 0.5% at a 1160 nm wavelength of light. When measuring the response time through photogating, the device achieved a GHz bandwidth due to the internal electric field and the drift speed.

### 2.5. Photodetector

In the aforementioned paper, investigations were conducted on the application of a split-gate in a photodetector. This involved altering the internal semiconductor doping state or flow targeted carriers.

**Figure 5:**
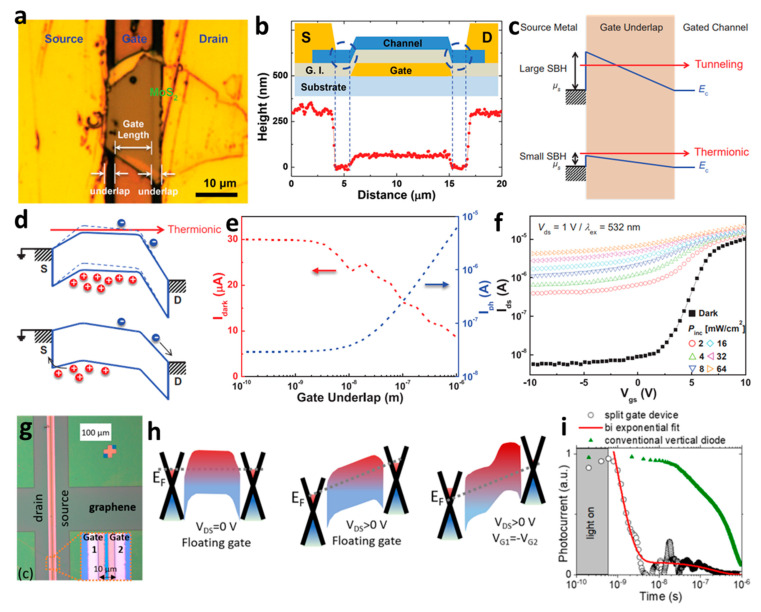
(**a**) Optical microscopy image age of a gate length shorter than the channel, (**b**) underlap section observed by AFM line scan. (**c**) Tunneling in the device caused by the underlap section. (**d**) Diagram of tunneling when light is irradiated, (**e**) dark current and photocurrent amplified by tunneling, (**f**) drain current by light intensity power [82] (adapted from [82] with permission from WILEY−VCH Verlag GmbH & Co. KGaA, Weinheim). (**g**) Optical microscopy image of a split-gate with a gap of 10 μm, (**h**) band bending according to the gate voltage and drain voltage, (**i**) efficient PN junction device implemented as a split −gate operating at high speed [84] (adapted from [84] with permission from American Chemical Society).

Although Kwon et al. did not employ a split-gate structure, they introduced a concept involving the creation of an underlap area on both sides of the gate at the bottom of the channel. This was achieved by designing the gate length to be shorter than the channel length [82]. The device used exfoliated MoS_2_ as the active semiconductor, as shown in Figure 5a. An underlap section was formed on both sides of the gate, as shown in the atomic force microscopy (AFM) profiling in Figure 5b. The gate underlap experienced less susceptibility to light irradiation and electrical signal application, resulting in reduced interference and influence on the gates. Hence, this design helps to minimize the interference from carriers (electrons and holes) generated by light, thereby enhancing the photocurrent. In the dark state, the device operates in a conventional *n*-channel mode. Due to the barrier in the gate underlap, current flows through electrons. However, when light is irradiated onto the phototransistor, it reaches the gate underlap, allowing the electron–hole pairs generated by the light to transport through the gate underlap. At this time, electrons and holes transmit through the energy barrier to transport, and thermionic tunneling is proposed, as shown in Figure 5c. As shown in Figure 5d, electrons are transported, and thermionic tunneling in the gate underlap plays a role in amplifying the photocurrent when light is irradiated. The electron–hole pairs generated by light are transported through the gate underlap, amplifying the current. As the series resistance varies depending on the length of the gate underlap, the current value also tends to change, and the results are shown in Figure 5e. In addition, the photocurrent according to power at 532 nm light, which is the wavelength at which light absorption of MoS_2_ occurs, and dark current are presented, as shown in Figure 5f.

Joshi et al. leveraged the photogating effect of the split-gate to control the electrical characteristics of the device [83]. Photogating involves controlling the conductance of a channel using a gate field caused by light. Photogating performs the gate effect by accumulating the photo-induced charge carriers at the interface between the gate and the semiconductor. Photogating activates the gate by reacting to the light. Photogating is used as a split-gate, and the light is screened by the gate at the top, so that the light is irradiated only to a specific area by controlling the location of the light. In addition, the charge concentration of the split-gate varies depending on the charge injection. Therefore, in this paper, the work function of the channel was adjusted to modulate the conductivity of the channel to improve the response to light.

Moreover, there is a study that achieved a fast photoresponse by applying a split-gate. Gréboval et al. employed a split-gate based on two split bottom gates separated by a length of 10 μm, as shown in Figure 5g [84]. As shown in Figure 5h, in the symmetric mode, in which the same gate voltage is applied, the same doping is caused at the junction, resulting in symmetrical current characteristics and a linear curve in the I–V relationship. On the other hand, in the opposite polarity gate bias, characterized by the application of non-identical gate bias, doping is induced under the opposite polarity voltage. When the opposite polarity voltage is applied, the split-gate establishes a lateral PN junction. In this mode, the PN junction exhibits a rectifying effect, resulting in a current when exposed to light. Due to this effect, gate bias can contribute two different effects: (i) modulating the Schottky barrier height at the graphene/HgTe interface, and (ii) generating a volume induction between the two gates at the PN junction. By expanding the volume of the junction, the device can capture more photons, generate additional carriers, and achieve higher photocurrents. Remarkably, the device exhibits a high efficiency of 100% EQE. Increasing the volume of the junction not only enhances device performance but also enlarges the light-sensitive area. Consequently, the application of the split-gate proves instrumental in facilitating charge separation, while HgTe nanocrystals contribute to efficient charge transport and separation. This enables the rapid capture and separation of photons, resulting in an enhanced photoresponse. Illustrated in Figure 5i, the decay time corresponding to a 90–10% reduction in the signal is 3 ns, demonstrating high-speed operation. The device achieves high-speed operation, as evidenced by a 3 dB cut-off frequency of 110 MHz. This demonstrates its capability to operate efficiently at high frequencies.

Mennel et al. employed the device for neuromorphic and image sensor functionalities, achieving high-speed operation through the introducing of the split-gate [85]. The active material chosen for this application is the representative ambipolar TMD, WSe_2_. The split-gate is specifically designed with a 300 nm gap length in the planar structure. In the operation of the photodiode, the voltage biasing of split-gate is adjusted to modulate the photocurrent, thereby controlling the weights in the neural network. When each photodiode, equipped with a split-gate, is exposed to light, it generates a photocurrent in distinct regions of the laterally separated region. By adjusting the photoresponse of each part using a split-gate, the device can control the sensitivity of each photodiode. By accurately adjusting the sensitivity, the photocurrent can be controlled, and the process of this photocurrent is used to compute the post-neural network. Each semiconductor channel finely tunes the photoresponse through split-gate biasing, allowing the photodiode to modulate the neural network weight during the learning process. Hence, each individual photodiode gains the ability to control the photocurrent. This operation extends to the image sensor, where it contributes to the formation of an artificial neural network (ANN) dedicated to image processing and classification. Notably, the high-speed operation of this system enables the processing of 20 million images per second, facilitating real-time operation where images are detected and processed simultaneously. This stands in contrast to conventional approaches that involve digital conversion before feeding data to a neural network. In this paper, the device was manufactured as a photodiode array, allowing for the real-time detection of images and the adjustment of the photoresponse matrix for each pixel and subpixel. In this process, the neural network functions as a weight, controlling the photocurrent of the image sensor photodiode through a photoresponse. Each photodiode updates the weight associated with each image, adapting dynamically during the learning process. Utilizing the neural network proposed by the authors, the image sensor itself becomes a powerful tool for processing and recognizing images, offering an efficient combination of speed and energy utilization.

### 2.6. High-Gain Amplifying Device

Using a split-gate allows the charge concentration inside the channel to be changed and the Fermi level to be adjusted, enabling its application in various scenarios. The modulation of the channel interior through gate voltage adjustment facilitates the tuning of linear output, making it suitable for use in amplification devices that leverage linear characteristics.

**Figure 6:**
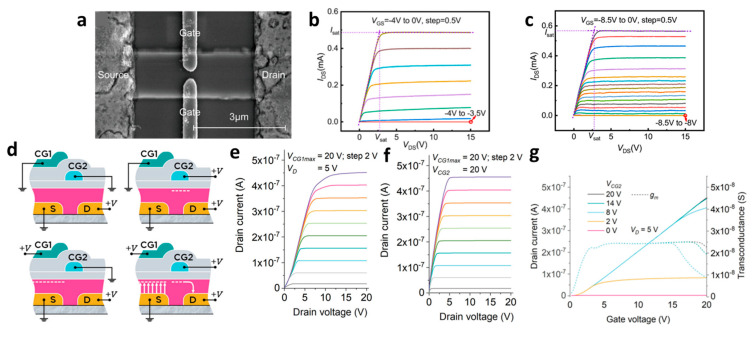
(**a**) Optical microscopy image displaying a submicron gap in an AlGaN/GaN heterostructure FET. (**b**) Output curve from a single−gate device without a split-gate. (**c**) Output curve from a device with a split-gate adopted [86] (adapted from [86] with permission from Elsevier B.V.). (**d**) Schematic diagram illustrating carrier generation when voltages are applied to each gate in a multi−modal transistor. (**e**) Transfer curve obtained by applying a fixed voltage to CG1 and sweeping the gate range with CG2. (**f**) Output curve where the value of CG2 is fixed, and it turns on linearly when the voltage is increased by 2 V in CG1. (**g**) Transfer curve and transconductance obtained by applying a fixed voltage to CG2 and sweeping the gate range with CG1 [87] (adapted from [87] with permission from The Society for Information Display).

Liu et al. engineered a split-gate device by constructing two gates, with a gap of 0.46 μm using UV lithography, as shown in Figure 6a [86]. The split-gate operation involved the application of the gate voltage, and it was observed in Figure 6b,c that the channel current changed as the gate voltage changed. In this structure, *I*_D_ is governed by polarization Coulomb field (PCF) scattering and a gate fringe electric field. By combining the gate fringe electric field and PCF scattering, the density of two-dimensional electron gas (2DEG) is reduced, leading to a corresponding decrease in mobility. As a consequence, a smaller *I*_D_ as the gate voltage becomes smaller, showing that the channel conductivity can be controlled with gate voltage. PCF scattering leverages the negatively polarized electrons within the channel, including scattering when a negative gate voltage is applied. When comparing Figure 6b,c, it is observed that between the outputs of the two devices, Figure 6c, using a split-gate, higher linearity, is achieved, while Figure 6b is by using a single gate. The use of a split-gate enables high-linearity voltage amplification by a larger input range and lower power consumption compared to the single gate. As a result, with the adoption of split-gate, the voltage gain (*g*_m_) reaches an amplification value of 14. This signifies that when a split-gate is adopted in a transistor, it becomes feasible to implement a common-source voltage amplifier effectively, enhancing the amplification of high-output signals concerning input signals.

An amplifier device using MMT, which has a similar structure to the split-gate, has been reported. Bestelink et al. proposed MMT using two control gates (CG) [87]. CG1 is responsible for regulating the current magnitude, while CG2 performs transportation or blocks the flow of charge. In the MMT structure, an intentional energy barrier is introduced to the source electrode to enable controlled current induction. The current is modulated through CG1, and the conductivity of the semiconductor region is independently adjusted through CG2. A constant transconductance is proposed, wherein the output current changes linearly with variations in CG1. As shown in Figure 6d, applying a positive bias to CG1 lowers the energy barrier, resulting in an increase in current, while a negative bias increases the energy barrier, leading to a decrease in *I*_D_. CG1 alters the output current. CG2 allows current to flow in the semiconductor when a positive bias is applied and blocks the current when a negative bias is applied. Therefore, the leakage current is minimized, operating at low power. The device exhibits a flat curve when turned on, in Figure 6e, under low or negative voltage applied to CG2. It was observed that the semiconductor conductivity decreases rapidly, interrupting the current flow. In contrast, by modulating the gate voltage through an increase in the voltage of CG2, it became feasible to achieve a flat saturation curve by controlling the contact using the potential barrier on the source side. Consequently, this device has demonstrated applicability in low-power scenarios and the realization of high-gain amplification devices (Figure 6f). Hence, as shown in Figure 6g, the linear dependence of *I*_D_ on the CG1 voltage is shown, with the corresponding conductance *g*_m_ is shown to adjust the linearity of the device according to the voltage adjustment of CG2. Therefore, this paper presents the possibility of analog signal processing utilizing the linear operation of MMT.

## 3. Conclusions and Outlook

In this paper, we introduced the structure and functionality of split-gates, which are utilized in neuromorphic systems, logic circuits, light-emitting transistors and diodes, photodetectors, and high-gain amplifiers. The operation of the device has been systematically validated based on the voltage polarity and value to separated gates.

In neuromorphic devices, the control of two gates functions as specific tasks, such as trapping/de-trapping carriers, regulating excitatory and inhibitory responses, and modulating the number of input pulses. In particular, some studies have demonstrated high-speed and low-voltage operation by mitigating the residual current caused by pre-synapse operation, which can interfere with post-synapse operations, through the application of an additional field. Logic circuits were introduced that were evaluated by modulating drain voltage and an operation mechanism according to the gate voltage range. The implementation of up to seven logic gates was achieved by expanding the combinations of drain voltage applied to each gate. Also, the capability to control individual carriers with separated gates has led to the development of LET, which can enable light emission at specific locations by injecting electrons and holes, respectively, and LED, which efficiently controls the Fermi level to implement an effective junction. Additionally, split-gate technology was also used in photodetectors, where devices have been introduced to enhance quantum efficiency by modifying the internal junction to capture more photons, resulting in higher a photocurrent. Also, the photogating effect in photodetectors, achieved by adjusting the gate, increases channel conductance through additional gates. And, finally, we introduced amplifier devices, which can prevent a specific field, modulate the Fermi level via controlled gate voltage, and achieve amplification operation with high linearity. The papers introduced in this manner are systematically arranged by application in Table 2. The table is organized into crucial parameters serving as significant indicators in the split-gate, including the split-gate structure in the device, the method for creating the gap within the split-gate, and the operational voltage at the split-gate. This paper highlights the broad utility of split-gates across various electronic and optoelectronic applications, underscoring their potential in advancing diverse fields of technology.

Split-gate technology applied to various fields can be adopted regardless of various types of materials and structures, suggesting the possibility of application in device fields that require the control of only the wanted carrier or the precise control of electrical characteristics. As technology has evolved, it has been observed that there is a widening scope of material utilization, the introduction of various gap patterning methods, and the emergence of reports highlighting devices with lower operating powers. However, for practical applications of split-gate devices, it is crucial to develop more straightforward and efficient split-gate devices, rather than relying on costly, ineffective lithography equipment-based techniques to manufacture thinner gap lengths. Therefore, cost-effective and high-yield manufacturing technologies such as lift-off lithography and adhesive lithography are necessitated. The fabrication of split-gate devices is predominantly restricted to robust substrates like Si or glass. To meet the diverse requirements of future applications, it is essential to expand manufacturing capabilities to include various form factors, such as flexible and stretchable substrates. The development of split-gate devices with different form factors is crucial for their widespread utilization in future applications. Furthermore, the stability of devices utilizing split-gate technology is not extensively reported. To ensure practical use, it is imperative to investigate its long-term stability and various stability characteristics under various operating conditions and modes.

## Figures and Tables

**Table 1:** Development of device application using split-gate by timeline.

Year	Device Type	Gap Length	Active Material	Dielectric Material	Operation Voltage	Ref.
1989	Split-gate field effect transistor	N/A	AlGaAs/GaAs	Undoped GaAs	V_th_ = −0.5 V	[47]
2006	Split-gate logic gate device	4 μm	Pentacene	SiO_2_	V_th_ = −4 V	[59]
2006	Split-gate high-gain amplifier device	400 nm	AlGaAs/GaAs	Undoped GaAs	V_th_ = −0.918 V	[60]
2008	Split-gate light-emitting transistor	1 μm	DCM doped Alq_3_	SiO_2_	V_to_ < 10 V	[61]
2014	Split-gate photodetector	1 μm	CNT	Al_2_O_3_	N/A	[62]
2018	Split-gate neuromorphic device	35 nm	Si	SiO_2_/Si_3_N_4_/SiO_2_	N/A	[63]

**Table 2:** Electronic devices that form a split-gate structure.

Application	Structure	Structure Image	Gap Length	Gap Patterning Method	Active Layer	Operation Voltage	Year	Ref.
N/A	Coplanar	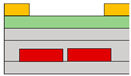	4 μm	Photolithography	PCDTBT: PC_70_BM	N/A	2010	[64]
	Coplanar	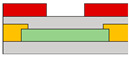	2 μm	Electron beam lithography	Carbon nanotube	N/A	2013	[67]
	Coplanar	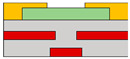	2.5 μm	Photolithography	C_10_-DNTT	V_th_ = 8 V	2014	[68]
	Coplanar	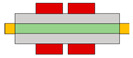	6 nm	N/A (Simulation)	Carbon nanotube	N/A	2020	[65]
	Vertical	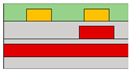	200 nm	Dielectric spacer	PNDTI-BT-DP	V_th,h_ = −17.7 V, V_th,e_ = 22.2 V	2018	[66]
Logic gate	Coplanar	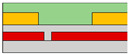	1 μm	Dry metal etching	DPP-TT-T	N/A	2016	[70]
	Vertical	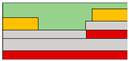	100 nm	Dielectric spacer	PDPP3T	V_on,h_ ≈ − 20 V, V_on,e_ ≈ 0 V	2016	[73]
	Vertical	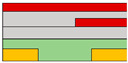	1 μm	Dielectric spacer	PDPP3T	N/A	2018	[69]
	Coplanar and vertical	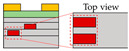	N/A & 100 nm	Photolithography and dielectric spacer	*µ*-Si	N/A	2021	[75]
	Coplanar	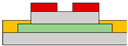	200 nm	Nanowire lift-off	PdSe_2_	V_th,p_ = −1.27 V, V_th,n_ = 1.80 V	2022	[71]
	Coplanar	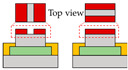	100 nm	Nanowire lift-off	MoS_2_	V_th,n_ ≈ 0 V, V_th,p_ ≈ −3 V	2022	[72]
Neuromorphic	Coplanar	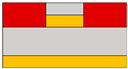	35 nm	Lithography	Si	N/A	2018	[63]
	Coplanar	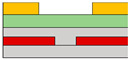	500 nm	Electron beam lithography	WSe_2_	N/A	2020	[79]
	Coplanar	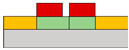	10 nm	N/A (Simulation)	SiGe	N/A	2022	[76]
	Coplanar	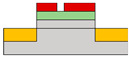	N/A	N/A	Hf_0.5_Zr_0.5_O_2_	N/A	2022	[77]
	Vertical	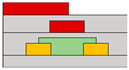	100 nm	Dielectric spacer	*µ*-Si	N/A	2022	[78]
Light-emitting transistor	Coplanar	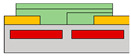	1 μm	Photolithography	DCM doped Alq_3_	V_on_ < 10 V	2008	[61]
	Coplanar	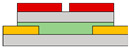	400 nm	Electron beam lithography	MoTe_2_	Diode threshold: 0.3 V (−0.3 V)	2017	[81]
	Vertical	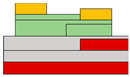	125 nm	Dielectric spacer	Alq_3_: DCJTB	V_th,n_ ≈ 40 V, V_th,p_ ≈ −19 V	2019	[80]
Photodetector	Coplanar (effect)	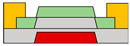	N/A	Split-gate effect by underlap	MoS_2_	V_th_ = 4.75 V	2015	[82]
	Vertical	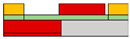	0.64 nm	Semiconductor spacer	MoTe_2_	N/A	2020	[83]
	Coplanar	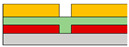	300 nm	Electron beam lithography	WSe_2_	N/A	2020	[85]
	Coplanar	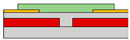	3 μm	Thermal evaporation	HgTe	N/A	2021	[84]
High-gain amplifier	Vertical	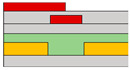	100 nm	Dielectric spacer	*µ*-Si	N/A	2020	[87]
	Coplanar	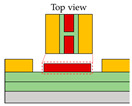	0.24 μm	Electron beam lithography	AlGaN/GaN	N/A	2022	[86]